# Pet Exposure Is Associated with Altered Gut Microbiota and Higher Phospholipid and Protein Concentrations in the Breast Milk of Overweight/Obese Pregnant Women

**DOI:** 10.3390/metabo16050317

**Published:** 2026-05-09

**Authors:** Yanpin Liu, Di Yang, Junying Zhao, Yan Liu, Yaling Wang, Yan Liu, Qian Liu, Xiaofei Fan, Bin Liu, Minghui Zhang, Weicang Qiao, Man Li, Jianyu Wang, Mengjing Du, Ling Guo, Lijun Chen

**Affiliations:** 1 College of Food Science, Northeast Agricultural University, Harbin 150030, China; liuyanpin@sanyuan.com.cn (Y.L.); di.yang@akesobio.com (D.Y.); fanxiaofei@ipsunshine.com (X.F.); 2 National Engineering Research Center of Dairy Health for Maternal and Child, Beijing Sanyuan Foods Co., Ltd., Beijing 100163, China; zhaojunying@sanyuan.com.cn (J.Z.); liuyan@sanyuan.com.cn (Y.L.); wangyaling@sanyuan.com.cn (Y.W.); liuyanlynn@cau.edu.cn (Y.L.); liuqian@sanyuan.com.cn (Q.L.); liubin@sanyuan.com.cn (B.L.); zhangminghui@sanyuan.com.cn (M.Z.); qiaoweicang@sanyuan.com.cn (W.Q.); liman989826@163.com (M.L.); jszxyjs@sanyuan.com.cn (J.W.); dumengjing@sanyuan.com.cn (M.D.); 3 Beijing Engineering Research Center of Dairy, Beijing Technical Innovation Center of Human Milk Research, Beijing Sanyuan Foods Co., Ltd., Beijing 100163, China

**Keywords:** breast milk, gut microbiota, maternal pet exposure, overweight/obese pregnant women

## Abstract

**Background:** The first 1000 days of a child’s life, from a woman’s pregnancy to her child’s second birthday, represent a critical window during which nutritional and environmental exposures shape long-term health. Gut microbiota play an important role in metabolic and overall health. Although pet exposure during pregnancy affects neonatal microbiota, immunity, and development, its effects on maternal health remain unclear. This study investigated the associations of pet exposure with gestational health, maternal and infant microbiota, and breast milk composition in overweight/obese pregnant women. **Methods**: Fecal samples and breast milk samples were collected from pet-exposed participants (*n* = 22) and non-exposed controls (*n* = 32) for 16S rRNA sequencing. Breast milk lipids and proteins were also quantified. **Results:** Pet exposure before conception, during pregnancy, and postpartum was not associated with gestational diabetes mellitus or gestational weight gain. In the maternal gut, the relative abundances of *Proteobacteria*, *Verrucomicrobia*, *Sutterellaceae*, *Enterobacteriaceae*, *Akkermansia muciniphila*, and *Parabacteroides* were higher, whereas that of Ruminococcus was lower, in the pet-exposed group. In breast milk, the relative abundance of *Escherichia-Shigella* and the concentrations of phosphatidylinositol 36:2, phosphatidylethanolamine 38:3, lysine, and β-casein were higher, whereas the abundance of *Rothia* was lower, in the pet-exposed group. The relative abundance of Escherichia-Shigella was also lower in the infant gut of the pet-exposed group. **Conclusions:** In overweight/obese pregnant women, pet exposure was associated with differences in maternal gut and breast milk microbiota, higher concentrations of selected breast milk phospholipids and β-casein, and lower *Escherichia-Shigella* abundance in the infant gut.

## 1. Introduction

The first 3 years of life are critical for gut microbiota development and are shaped by maternal health, environmental microbial exposure, and breast milk composition. The incidence of metabolic disorders associated with gut microbial dysbiosis, including gestational diabetes mellitus and maternal overweight or obesity, has increased in recent decades [1]. Maternal metabolic disorders adversely affect fetal growth and development and may increase the risk of metabolic syndrome and diabetes in the offspring [2].

Household pets, particularly cats and dogs, introduce new microbial taxa into the home environment and may alter microbial abundance, diversity, and dominant taxa [3,4]. Accumulating evidence indicates that pets and their owners can harbor overlapping gut microbial communities [5] and that regular contact with companion animals may modulate both the taxonomic composition and functional capacity of the human gut microbiota [6]. In a study of 218 newborns, greater household biodiversity, assessed by the number of fur-bearing pets, was associated with greater diversity of the neonatal fecal microbiota [7]. The growing number of pet-owning households worldwide has increased interest in the effects of maternal pet exposure during pregnancy on both maternal and infant health. The hygiene hypothesis, first proposed in 1989, suggests that exposure to pets may confer immune benefits, whereas an overly hygienic environment may increase the risk of allergic disease [8]. With growing evidence linking gut microbial dysbiosis to allergic disease, this concept has been refined into the “microbiological hypothesis” [9].

Cassidy-Bushrow et al. reported a twofold higher risk of obesity at 2 years of age in infants delivered by cesarean section than in those delivered vaginally [10]. However, this association was not observed among children whose mothers were exposed to pets during pregnancy. Maternal pre- or postnatal pet exposure has been associated with lower offspring IgE levels through 2 years of age and with a reduced risk of allergic diseases, including atopic dermatitis and asthma. In addition, postnatal pet exposure has been associated with a lower risk of metabolic disease in early life [11,12]. However, little is known about the associations of pet exposure before conception, during pregnancy, and postpartum with gestational weight gain (GWG), gestational diabetes mellitus (GDM), maternal microbiota, and breast milk composition.

We therefore hypothesized that maternal pet exposure would be associated with GWG, GDM, maternal gut microbiota, and breast milk composition in overweight/obese pregnant women. The primary objective was to evaluate the associations of maternal pet exposure with GWG and GDM, and the secondary objective was to assess its associations with maternal gut microbiota, breast milk microbiota, and breast milk composition. This study may provide a basis for understanding how maternal pet exposure is associated with maternal and infant health-related outcomes.

## 2. Materials and Methods

### 2.1. Study Participants

The eligibility criteria were age 18–35 years, singleton pregnancy, gestational age of 8–12 weeks at recruitment, residence in Beijing for at least 5 years, and no history of pre-pregnancy hypertension, diabetes, hyperlipidemia, hepatitis, nephritis, gastrointestinal disease (chronic gastritis, enteritis, or gastric/duodenal ulcer), or infectious disease (hepatitis or tuberculosis). The exclusion criteria were antibiotic use within the previous month, regular smoking or alcohol consumption, conception by assisted reproductive technology, inability to complete the questionnaire because of mental illness, and unwillingness to participate in the survey.

### 2.2. Grouping and Exposure Classification

Participant recruitment began on 15 March 2018. Eligible participants were identified through a questionnaire-based screening process. A total of 22 pregnant women who were exposed to pets before conception, during pregnancy, and postpartum, together with their infants, were assigned to the pet-exposed group, whereas 32 pregnant women without pet exposure, together with their infants, were assigned to the non-exposed group. Among the enrolled participants, 17 women in the pet-exposed group and 22 in the non-exposed group consumed fermented milk continuously during pregnancy and the postpartum period. In this study, fermented milk specifically refers to yogurt. Other fermented dairy products, such as kefir, koumiss, and fermented milk beverages, were excluded.

Because fermented milk intake during pregnancy may affect both maternal gut microbiota and breast milk microbiota, fermented milk consumption was included as a covariate in analyses evaluating the association between pet exposure and these microbial outcomes [13,14]. For subsequent analyses, the groups were defined as follows: pet exposure (P), no pet exposure (NP), pet exposure with fermented milk consumption (P-F), no pet exposure with fermented milk consumption (NP-F), pet exposure without fermented milk consumption (P-NF), and no pet exposure without fermented milk consumption (NP-NF). Information on pet exposure was obtained at recruitment, and additional medical data were extracted from hospital records. All participants completed a standardized questionnaire.

This study was approved by the Medical Ethics Committee of Beijing Obstetrics and Gynecology Hospital, Capital Medical University (No. 2017-KY-015-02), and all participants provided written informed consent. Written parental consent was obtained for infant sample collection.

### 2.3. Sample Collection and Clinical Data Sources

**Figure 1:**
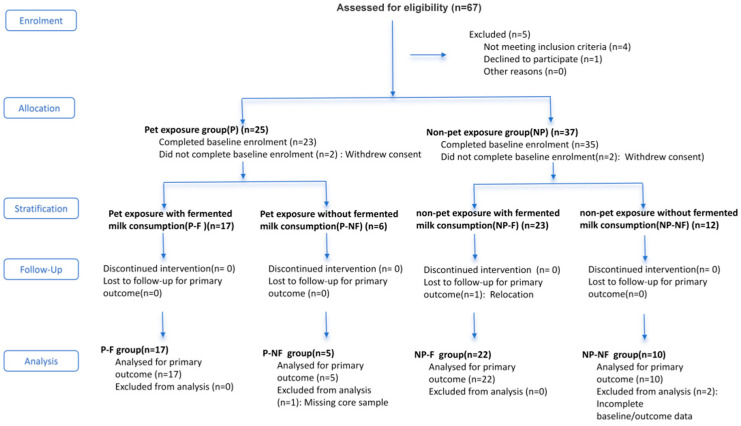
Study grouping.

Maternal fecal samples were collected during early pregnancy (gestational weeks 8–12), mid-pregnancy (gestational weeks 24–28), late pregnancy (gestational weeks 36–40), and at 42 ± 1 days postpartum. Infant fecal samples were collected at 6 months, and breast milk samples were collected at postpartum days 1–5 and 42 ± 1 and at postpartum months 3–6 and 6–12 (Figure 1). Maternal health follow-up forms were completed at each sampling time point. At home, participants collected stool samples from the toilet, immediately transferred them into collection tubes containing stool DNA stabilizer (Stratec, Birkenfeld, Germany), and stored them at −20 °C. Samples were transported to the laboratory within 24 h and then stored at −80 °C. After hand washing with soap or liquid detergent and cleaning the breast with a clean tissue, milk was expressed from one breast using a breast pump between 9:00 and 11:00 a.m. Foremilk and hindmilk were mixed thoroughly, and a 10 mL milk sample was transferred into a sterile centrifuge tube, stored temporarily at −20 °C, and moved to −80 °C storage within 24 h.

GDM was diagnosed in early pregnancy (mean gestational age, 14.6 weeks) or mid-pregnancy (mean gestational age, 26.3 weeks) according to national guidelines, as previously described [15]. GDM was diagnosed according to the American Diabetes Association criteria on the basis of a 75 g oral glucose tolerance test, with thresholds of 5.1 mmol/L for fasting plasma glucose, 10.0 mmol/L for 1 h glucose, and 8.5 mmol/L for 2 h glucose [16].

### 2.4. Fecal and Breast Milk Microbiota Analysis

Total genomic DNA was extracted from 100 mg of homogenized fecal samples using the TIANamp Stool DNA Kit (Tiangen, Beijing, China; catalog no. DP712) and from 5 mL of homogenized breast milk using the cetyltrimethylammonium bromide method. Parallel DNA extraction was also performed for all samples using the QIAamp Fast DNA Stool Mini Kit (Qiagen GmbH, Hilden, Germany) according to the manufacturer’s instructions.

DNA purity and concentration were assessed by 1% agarose gel electrophoresis, and all DNA samples were diluted to 1 ng/µL with sterile nuclease-free water. The V3–V4 hypervariable region of the 16S rRNA gene was amplified using barcoded primers 341F (CCTACGGGNGGCWGCAG) and 805R (GACTACHVGGGTATCTAATCC). Each 30 µL reaction contained 10 ng of template DNA, 15 µL of Phusion High-Fidelity PCR Master Mix (New England Biolabs, Ipswich, MA, USA), and 0.2 µM of each primer. Thermal cycling conditions were 98 °C for 1 min and 30 cycles of 98 °C for 10 s, 50 °C for 30 s, and 72 °C for 30 s, followed by a final extension at 72 °C for 5 min. PCR amplicons were visualized by 2% agarose gel electrophoresis, purified using the Universal DNA Purification Kit (Tiangen, China; catalog no. DP214), and used to construct sequencing libraries with the NEBNext Ultra II FS DNA PCR-Free Library Prep Kit (New England Biolabs, Ipswich, MA, USA; catalog no. E7430L) according to manufacturer protocol. Libraries were quantified by Qubit fluorometry and quantitative PCR, validated for fragment size using a Bioanalyzer, and sequenced on an Illumina platform.

For bioinformatic processing, raw reads were demultiplexed using sample-specific barcodes, trimmed to remove primer and barcode sequences, and merged into raw tags using FLASH (v1.2.11) [17]. Quality filtering was performed using Fastq (v0.23.1) to obtain clean tags [18], followed by chimera removal with the UCHIME algorithm against the SILVA and UNITE reference databases [19]. Effective tags were clustered into operational taxonomic units (OTUs) at 97% sequence similarity using UPARSE (v7.0.1001). Representative sequences for each OTU were taxonomically annotated against the SILVA 138.1 database.

### 2.5. Breast Milk Composition Analysis

#### 2.5.1. Breast Milk Lipid Analysis to Assess Inter-Sample Variation

The methods used to quantify breast milk lipids have been described previously [20]. Briefly, 200 µL of breast milk was spiked with internal standards, including phosphatidylcholine (PC), phosphatidylethanolamine (PE), sphingomyelin (SM), phosphatidylinositol (PI), phosphatidylglycerol (PG), ceramide (Cer), and triacylglycerol (TAG), as follows: 20 µL of 10.38 mg/L PC17:0-14:1, 20 µL of 9.64 mg/L PE17:0-14:1, 20 µL of 10.00 mg/L SM35:1, 8 µL of 10.22 mg/L PI17:0-14:1, 4 µL of 9.81 mg/L PG17:0-14:1, 4 µL of 10.00 mg/L Cer42:2, and 20 µL of 4000 mg/L d5-TAG54:3. Two sequential extraction steps were performed: the first used 200 µL of ultrapure water, 2 mL of methanol, and 900 µL of dichloromethane, and the second used 200 µL of ultrapure water and 900 µL of dichloromethane. The mixture was centrifuged at 3381× *g* for 15 min to separate the organic and aqueous phases. The organic phase was then combined with 1 mL of ultrapure water, 2.2 mL of methanol, and 600 µL of dichloromethane, vortexed thoroughly, and centrifuged at 3000× *g* for 10 min to separate the phases. The collected aqueous phase was re-extracted with 1.8 mL of dichloromethane and centrifuged at 3381× *g* for 15 min to achieve complete phase separation. The two organic phases were collected and dried under a stream of nitrogen. The dried sample was reconstituted in 1 mL of reconstitution solution. The glyceride-containing fraction was diluted 1000-fold, and 50 µL of the reconstituted sample was injected into the ultra-performance liquid chromatography/quadrupole time-of-flight mass spectrometry system for analysis. Kinetex 2.6 µm C18 100 Å columns (Phenomenex, Torrance, CA, USA) measuring 150 × 4.6 mm and 50 × 3 mm were used to separate phospholipids and triacylglycerols, respectively. The chromatographic conditions were as follows: injection volume, 2 µL; flow rate, 0.8 mL/min; and column temperature, 30 °C. Mobile phase A consisted of 5 mM ammonium acetate in water/methanol/acetonitrile (1:1:1, *v*/*v*/*v*), and mobile phase B consisted of 5 mM ammonium acetate in isopropanol.

#### 2.5.2. Breast Milk Fatty Acid Analysis

Fatty acids were methyl-esterified using the hydrochloric acid–methanol method. Briefly, 200 µL of breast milk was added to 5 mL of 0.5 mol/L hydrochloric acid–methanol solution, followed by 2 mL of n-hexane and 2 mL of methanol. The mixture was then incubated in an 80 °C water bath for 2 h with shaking. The tubes were then removed and cooled to 25 °C under running water. Next, 2 mL of deionized water was added, and the tubes were mixed thoroughly, frozen, and centrifuged at 2348× *g* for 5 min. A 1 mL aliquot of the ester-layer supernatant was then transferred to an injection vial for gas chromatography–mass spectrometry analysis. Samples were stored at −20 °C until analysis.

Gas chromatography–mass spectrometry analysis was performed using an HP-88 column (100 m × 0.25 mm, 0.20 µm) with the following temperature program: initial hold at 60 °C for 5 min, ramp to 160 °C at 8 °C/min, ramp to 200 °C at 4 °C/min with a 5 min hold, and final ramp to 240 °C at 3 °C/min with a 5 min hold. The gas chromatography–mass spectrometry system was equipped with a TriPlus RSH autosampler (Thermo Fisher Scientific Inc., Waltham, MA, USA). The inlet temperature was 200 °C, the injection volume was 1 µL, the split ratio was 10:1, and nitrogen was used as the carrier gas at a flow rate of 1 mL/min. An electron ionization source was used with the following operating parameters: ion source temperature, 280 °C; transfer line temperature, 240 °C; quadrupole temperature, 150 °C; and quadrupole mass scan range, approximately 35–400 *m*/*z* [21]. Fatty acid methyl esters were identified on the basis of their characteristic ions and retention times and quantified by comparing peak areas with those of fatty acid methyl ester standards of known concentration.

#### 2.5.3. Breast Milk Amino Acid Analysis

Amino acids were quantified by ultra-performance liquid chromatography (UPLC) using a photodiode array detector, an AccQ-Tag Ultra C18 column (1.7 µm, 2.1 × 100 mm; Waters Corporation, Milford, MA, USA), and an AccQ-Fluor reagent kit (Waters Corporation, Milford, MA, USA) for precolumn derivatization. A 300 µL breast milk sample was mixed thoroughly with 20 µL of 10% sulfosalicylic acid in a 1.5 mL centrifuge tube, centrifuged at 9408× *g* for 15 min at 4 °C, and filtered through a 0.22 µm membrane filter. A 10 µL aliquot of the filtrate was transferred to a UPLC vial and combined with 70 µL of 0.4 M borate buffer and 20 µL of AccQ-Fluor reagent. After incubation at 55 °C for 10 min, the mixture was cooled to room temperature before UPLC analysis. Standard solutions (2.5 mM) of 17 amino acids, including lysine, serine, and arginine, were processed in the same manner. A 1 µL aliquot of the derivatized sample was injected into the UPLC system and detected at 260 nm using a photodiode array detector [22]. The mobile phases consisted of AccQ-Tag Ultra Eluent A and AccQ-Tag Ultra Eluent B supplied by the manufacturer. The UPLC gradient program used the following solvents: A, AccQ-Tag Ultra Eluent A; B, 10% AccQ-Tag Ultra Eluent B; C, water; and D, AccQ-Tag Ultra Eluent B.

### 2.6. Statistical Analysis

Data analysis was performed using R software (v4.0.5; R Foundation for Statistical Computing, Vienna, Austria). The operational taxonomic unit (OTU) matrix was processed using the vegan package in R, and alpha diversity was calculated using the abundance-based coverage estimator, Chao1, and Shannon indices. To assess inter-sample variation in community composition, unweighted UniFrac distances were calculated from the OTU matrix in R, followed by principal coordinate analysis (PCoA) [23]. Intergroup comparisons of median richness, diversity, and the relative abundance of dominant taxa were performed using the nonparametric Kruskal–Wallis test. After conversion of the OTU matrix to a relative abundance matrix, linear discriminant analysis effect size was performed using the microeco package (v0.6.0) in R to identify taxa with significant intergroup differences, with a linear discriminant analysis score threshold of ≥2.0 and a significance level of *p* < 0.05. The microbiota of samples from the pet-exposed and non-exposed groups were then compared using the Mann–Whitney U test. Multivariable logistic regression was used to assess the independent association between microbial abundance and pet exposure. Phylogenetic Investigation of Communities by Reconstruction of Unobserved States 2 analysis was performed using the Kyoto Encyclopedia of Genes and Genomes (KEGG) pathway database to predict microbial functions [24]. A rank-sum test in SPSS Statistics 26.0 was used to compare breast milk composition between the two groups. Spearman’s rank correlation coefficient was used to evaluate the associations of pet exposure and other covariates with microbiota composition and to assess correlations between specific gut microbial taxa, GDM, and breast milk components in the two groups.

## 3. Results

### 3.1. Study Population and Household Pet Exposure

Among the 54 overweight/obese pregnant women, 22 (40.7%) had pet exposure from before conception through the postpartum period; of these, 10 (45.5%) had cats only, 14 (63.6%) had dogs only, and 2 (9.1%) had both cats and dogs. Among the pet-exposed women, 17 (77.3%) consumed fermented milk continuously from the first trimester through the postpartum period. By contrast, 32 women (59.3%) had no pet exposure, of whom 22 (68.7%) consumed fermented milk continuously from the first trimester through the postpartum period. Table 1 summarizes maternal characteristics according to pet exposure status. Pet exposure status differed significantly according to maternal age (*p* = 0.05), fermented milk intake (*p* = 0.04), and breastfeeding status (*p* = 0.002).

### 3.2. Gestational Diabetes Mellitus and Gestational Weight Gain

The oral glucose tolerance test (OGTT) was administered at a mean gestational age of 24.9 weeks. The prevalence of GDM was 17.6%, 13.6%, 0.0%, and 30.0% in the P-F, NP-F, P-NF, and NP-NF groups, respectively. The prevalence of GDM did not differ significantly among the groups. Fasting, 1 h post-OGTT, and 2 h post-OGTT glucose levels were lower in the pet-exposed groups than in the non-exposed groups regardless of fermented milk consumption, although the differences were not statistically significant (*p* > 0.05; Table 2).

Because GWG is closely associated with GDM, we also examined the association between pet exposure and GWG. GWG was higher in the P-F group than in the NP-F group but lower in the P-NF group than in the NP-NF group (Figure S1). However, none of these between-group differences were statistically significant (*p* > 0.05). According to the Monitoring and Evaluation of Gestational Weight in Chinese Women report, the recommended GWG range is 7.0–11.0 kg for overweight women (24.0 kg/m^2^ < BMI < 28.0 kg/m^2^) and 5.0–9.0 kg for obese women (BMI ≥ 28 kg/m^2^). In the P-F and NP-F groups, 70.6% and 40.9% of women, respectively, exceeded the recommended GWG range. Similarly, 40% of women in both the P-NF and NP-NF groups exceeded the recommended GWG range, with no significant between-group difference (*p* > 0.05).

### 3.3. Maternal Gut Microbiota According to Pet Exposure

#### 3.3.1. The Group That Consumed Fermented Milk

**Figure 2:**
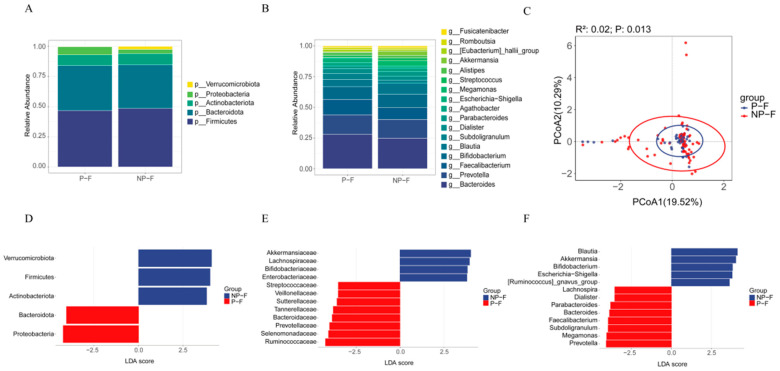
Maternal gut microbiota composition in the P-F and NP-F groups. Relative abundance of maternal gut microbiota at the (**A**) phylum and (**B**) genus levels. (**C**) Principal coordinate analysis (PCoA) of the P-F and NP-F groups at the OTU level. Linear discriminant analysis (LDA) score distribution between the P-F and NP-F groups at the (**D**) phylum, (**E**) family, and (**F**) genus levels.

Microbial composition analysis showed that Firmicutes, Bacteroidetes, Actinobacteria, and Proteobacteria were the predominant phyla in both the P-F and NP-F groups (Figure 2A). At the genus level, *Bacteroides*, *Prevotella*, *Faecalibacterium*, and *Bifidobacterium* were the predominant taxa (Figure 2B). However, gut microbiome composition differed significantly at the OTU level (PCoA, *p* = 0.01; Figure 2C). Compared with the NP-F group, the P-F group had a higher relative abundance of Proteobacteria at the phylum level (Figure 2D and Table S1), Barnesiellaceae, Ruminococcaceae, and Sutterellaceae at the family level (Figure 2E and Table S1), and *Barnesiella* at the genus level (Figure 2F and Table S1).

#### 3.3.2. Participants Who Did Not Consume Fermented Milk

**Figure 3:**
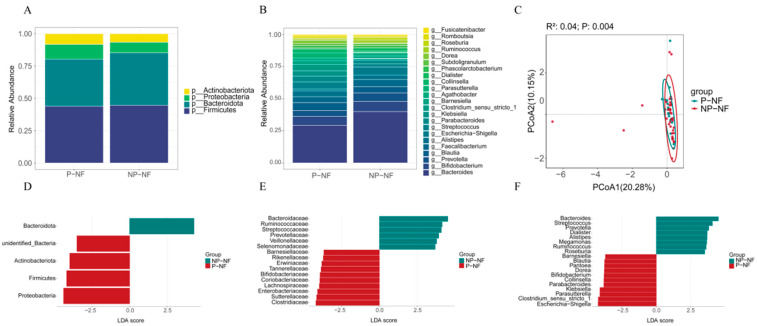
Maternal gut microbiota composition in the P-NF and NP-NF groups. Relative abundance of maternal gut microbiota at the (**A**) phylum and (**B**) genus levels. (**C**) Principal coordinate analysis (PCoA) of the P-NF and NP-NF groups at the OTU level. Linear discriminant analysis (LDA) score distribution between the P-NF and NP-NF groups at the (**D**) phylum, (**E**) family, and (**F**) genus levels.

Firmicutes, Bacteroidota, Proteobacteria, and Actinobacteriota were the predominant phyla across all samples (Figure 3A). At the genus level, *Bacteroides*, *Bifidobacterium*, *Prevotella*, *Blautia*, *Faecalibacterium*, *Alistipes*, *Escherichia-Shigella*, and *Streptococcus* were the dominant taxa (Figure 3B). Alpha diversity analysis showed no significant between-group differences in microbiota richness (Chao1 and ACE, *p* > 0.05) or diversity (Shannon index, *p* > 0.05) between the P-NF and NP-NF groups (Figure S3). Microbiome composition differed significantly between the P-NF and NP-NF groups at the OTU level (*p* < 0.01; Figure 3C). Compared with the NP-NF group, the P-NF group had significantly higher relative abundances of Proteobacteria and Verrucomicrobia at the phylum level (Figure 3D); Clostridiaceae, Sutterellaceae, Enterobacteriaceae, and Akkermansiaceae at the family level (Figure 3E); and *Parabacteroides*, *Clostridium*, and *Akkermansia* at the genus level (Figure 3F). Conversely, the relative abundances of *Megamonas*, *Ruminococcus*, and *Veillonella* were significantly lower in the P-NF group than in the NP-NF group (Table S1). Gut microbiota abundance did not differ significantly between pet-exposed and non-exposed women during early, mid-, or late pregnancy (Table S2).

#### 3.3.3. Associations Between Pet Exposure and Maternal Gut Microbiota After Covariate Adjustment

To assess whether the association between pet exposure and microbial abundance was independent of measured covariates, adjusted logistic regression analysis was performed. After sequential adjustment for potential confounders listed in Table 1, including fermented milk consumption, maternal age at pregnancy, and preconception weight, the associations between pet exposure and maternal gut microbiota were generally unchanged, except that *Megamonas* abundance was associated with gestational age (*p* = 0.03).

#### 3.3.4. Predicted Functional Profiles of the Maternal Gut Microbiome According to Pet Exposure

**Figure 4:**
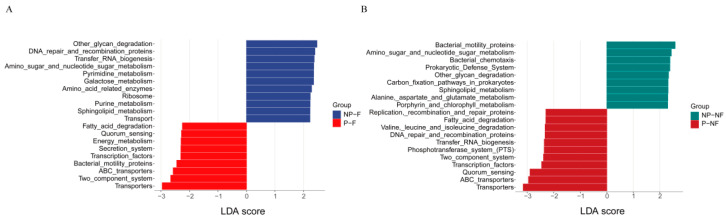
Predicted metagenomic functions based on Kyoto Encyclopedia of Genes and Genomes (KEGG) pathway analysis. Extended bar plots show significantly different KEGG pathways between the (**A**) P-F and NP-F groups and (**B**) P-NF and NP-NF groups.

A total of 386 KEGG pathways were predicted from the OTUs identified in the P-F and NP-F groups, and 383 KEGG pathways were predicted from the OTUs identified in the P-NF and NP-NF groups (Figure 4). The P-F group showed lower predicted enrichment of several disease-associated pathways, including hyperlipidemia, diabetes, and oxidative stress, whereas the NP-NF group showed lower predicted enrichment of type 2 diabetes mellitus and obesity pathways.

### 3.4. Breast Milk Composition According to Pet Exposure

#### 3.4.1. Breast Milk Microbiota According to Pet Exposure

**Figure 5:**
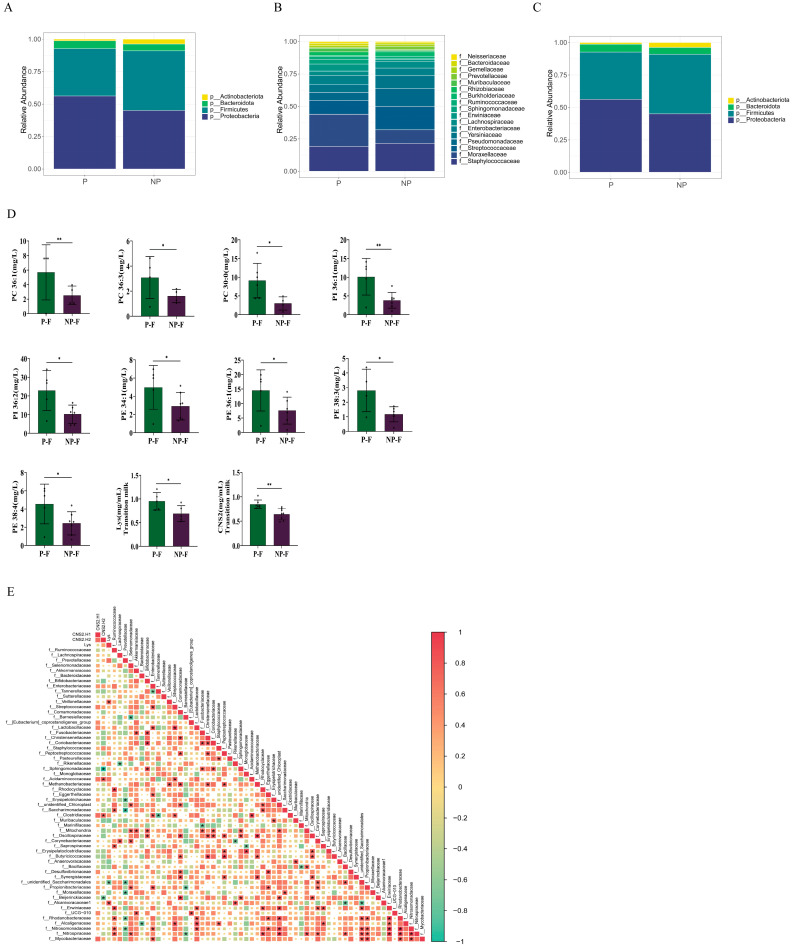
Microbiological and biochemical composition of breast milk. Relative abundance of breast milk microbiota in the P and NP groups at the (**A**) phylum, (**B**) family, and (**C**) genus levels. (**D**) Differences in breast milk lipid and protein composition. (**E**) Spearman correlation analysis between maternal gut microbiota and breast milk components. * *p* < 0.05, ** *p* < 0.01.

Because limited breast milk was collected at each postpartum stage, samples from all lactation stages were combined for analysis. Pet exposure before conception, during pregnancy, and postpartum was not associated with significant differences in breast milk microbial richness (Chao1 and ACE, *p* > 0.05; Figure S4), diversity (Shannon index, *p* > 0.05; Figure S4), or overall composition (*p* > 0.05; Figure S5). At the phylum level, the relative abundances of Proteobacteria and Bacteroidetes were lower in the P group than in the NP group, although the differences were not statistically significant (*p* > 0.05; Figure 5A). At the family level, dominant microbiota composition did not differ significantly between the P and NP groups (Figure 5B and Table S3). At the genus level, the relative abundance of *Rothia* was significantly lower in the P group than in the NP group (*p* = 0.04; Figure 5C and Table S3), whereas the relative abundance of *Escherichia-Shigella* was significantly higher in the P-NF group than in the NP-NF group (*p* = 0.03; Table S3). However, median breast milk microbiota abundance did not differ significantly between the P-F and NP-F groups.

#### 3.4.2. Breast Milk Lipid and Protein Composition According to Pet Exposure

Lipids, proteins, and fatty acids were quantified in colostrum, transitional milk, and mature milk from the P-F and NP-F groups. Sixteen phospholipids were significantly more abundant in colostrum from the P-F group than in colostrum from the NP-F group (*p* < 0.05; Figure 5D), including PC 36:1, PC 36:3, PC 30:0, PI 36:1, PI 36:2, PE 34:1, PE 36:1, PE 38:3, and PE 38:4. Twenty amino acids were quantified in transitional milk, and lysine concentrations were significantly higher in the P-F group than in the NP-F group (*p* < 0.05; Figure 5D). Eight proteins—lactoperoxidase, immunoglobulin M, immunoglobulin G, κ-casein, immunoglobulin A, lactotransferrin, β-casein, and secretory immunoglobulin A—were quantified in transitional milk; β-casein concentrations were significantly higher in the P-F group than in the NP-F group (*p* < 0.05; Figure 5D). Thirty-seven fatty acids were identified and quantified in colostrum, with no statistically significant differences between the P-F and NP-F groups (*p* > 0.05).

#### 3.4.3. Correlations Between Differential Microbiota and Breast Milk Components

Spearman’s correlation analysis was performed to assess associations between differential maternal gut microbiota and differential breast milk lipid and protein components. The abundance of Sutterellaceae was positively correlated with PE 34:1 and PE 38:3 concentrations (Figure 5E). Consistently, Sutterellaceae abundance and PE 34:1 and PE 38:3 concentrations were significantly higher in the P-F group than in the NP-F group.

### 3.5. Infant Gut Microbiota According to Pet Exposure

**Figure 6:**
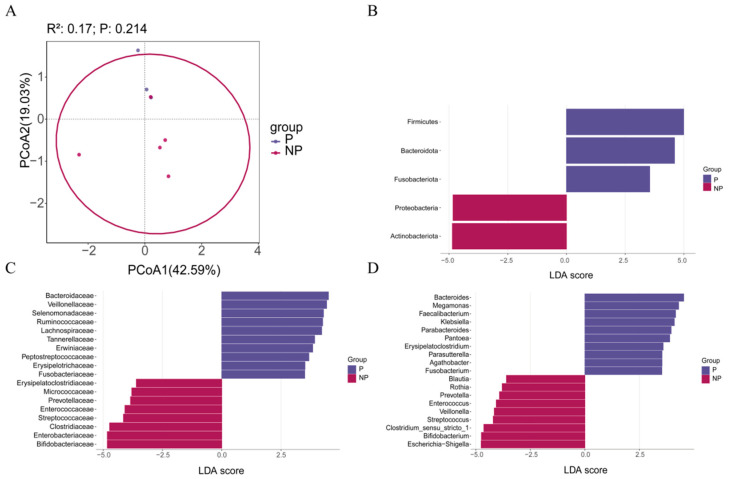
Infant gut microbiota composition according to maternal pet exposure. (**A**) Principal coordinate analysis (PCoA) of the P and NP groups at the OTU level. Linear discriminant analysis (LDA) score distribution between the P and NP groups at the (**B**) phylum, (**C**) family, and (**D**) genus levels.

Overall infant gut microbiome diversity (Shannon index, *p* > 0.05; Figure S6), richness (Chao1 and ACE, *p* > 0.05; Figure S6), and composition (PCoA, *p* > 0.05; Figure 6A) did not differ significantly between the P and NP groups. Linear discriminant analysis effect size (LEfSe) showed that Firmicutes and Bacteroidota at the phylum level and Ruminococcaceae and Oscillospiraceae at the family level were significantly more abundant in the P-NF group (*p* < 0.05; Figure 6B,C), whereas *Escherichia-Shigella* and *Bifidobacterium* at the genus level were significantly more abundant in the NP-NF group (*p* < 0.05; Figure 6D). The Enterobacteriaceae/Bacteroidaceae ratio (E/B ratio) was lower in the pet-exposed group, but the difference was not statistically significant (*p* > 0.05).

## 4. Discussion

### 4.1. Principal Findings

In this cohort of 54 overweight/obese pregnant women, maternal pet exposure was not associated with the prevalence of GDM or with GWG. However, pet exposure was associated with higher relative abundances of Verrucomicrobia, Sutterellaceae, *Ruminococcus*, and *Akkermansia muciniphila* in the maternal gut and with higher breast milk concentrations of PI 36:2, PE 38:4, lysine, and β-casein. By contrast, pet exposure was associated with a lower relative abundance of *Escherichia-Shigella* in the infant gut. To our knowledge, this is the first study to examine associations between maternal pet exposure and maternal gut microbiota, breast milk composition, and infant gut microbiota in overweight/obese pregnant women.

### 4.2. Maternal Gut Microbiota and Metabolic Relevance

Maternal pet exposure was associated with a lower relative abundance of *Ruminococcus*, a genus previously linked to obesity-related metabolic phenotypes [25]. In a study evaluating gut microbiota as a predictor of weight-loss trajectories, *Ruminococcus* was enriched in individuals with obesity, and its abundance decreased during weight loss [26]. Another study reported associations between *Ruminococcus* and obesity-related indicators, including fat mass, body mass index, waist circumference, C-reactive protein, and triglyceride levels. Notably, *Ruminococcus* has also been detected in cat and dog feces [27]. However, whether differences in maternal gut *Ruminococcus* abundance reflect microbial transfer from domestic pets requires further investigation.

In participants who did not consume fermented milk during pregnancy, maternal pet exposure was associated with a higher relative abundance of *Parabacteroides*, a common member of the human gut microbiota. Previous correlation analyses have linked *Parabacteroides* abundance inversely with obesity, nonalcoholic fatty liver disease, and diabetes [28]. Thus, *Parabacteroides* may be relevant to maternal glucolipid metabolism in this cohort, although this potential role requires mechanistic confirmation.

Maternal pet exposure was also associated with higher abundances of Verrucomicrobiota-related taxa, including *Akkermansia muciniphila*, in the maternal gut, particularly among women who did not consume fermented milk during pregnancy. These microbes can use mucin as an energy source in the gut mucus layer and may support mucus-layer thickness and gut-barrier integrity [29]. Human and mouse studies have reported lower *Akkermansia muciniphila* abundance in overweight or obese hosts than in healthy controls [30]. In probiotic-supplementation studies, *Akkermansia muciniphila* reduced plasma lipopolysaccharide-binding protein, leptin levels, liver and muscle LPS/LBP downstream signaling, fat deposition, glucose intolerance, and low-grade inflammation [31]. In a Dutch three-generation family study of 8208 participants from 2756 families, cohabitation was a major factor associated with gut microbial composition, function, antibiotic resistance, and virulence factors; pets were included in the cohabitation category, and 6.6% of microbial taxa, including *Akkermansia muciniphila*, were shared [32]. These findings suggest that pet exposure may be linked to maternal gut *Akkermansia muciniphila* abundance, but whether this association influences infant microbial colonization or later obesity risk remains unknown.

### 4.3. Breast Milk Microbiota and Infant Gut Microbiota

Breast milk is a key determinant of early intestinal development and immune maturation in infants. Maternal pet exposure was associated with a higher relative abundance of *Escherichia-Shigella* in breast milk. Because *Escherichia-Shigella* includes potentially pathogenic taxa, higher exposure through breast milk could plausibly influence intestinal immune activation and inflammatory signaling [33]. However, analysis of infant gut microbiota at 6 months showed that *Escherichia-Shigella* abundance was significantly lower in infants of pet-exposed mothers than in infants of non-exposed mothers. The P-NF group also had higher Firmicutes abundance and lower *Bifidobacterium* spp. abundance, a pattern that may reflect infant gut microbiota maturation.

One possible interpretation is that early microbial exposure through breast milk may contribute to immune priming and gut microbiota maturation [34]; however, this study did not assess infection outcomes or immune markers, so this hypothesis requires further testing. Additionally, the lower Enterobacteriaceae/Bacteroidaceae ratio in infants of pet-exposed mothers was consistent with the findings of Tun et al. [35]. The E/B ratio has been proposed as an indicator of gut microbiota maturity, and a higher ratio has been associated with allergy risk [36].

Breast milk analysis also showed that maternal pet exposure was associated with lower *Rothia* abundance. Because *Rothia* spp. can produce short-chain fatty acids that interact with G protein-coupled receptors in gut epithelial cells, this finding may be relevant to gut-barrier and immune regulation. Short-chain fatty acids can also modulate immune cells and pro-inflammatory mediators and have been linked to protection against inflammatory bowel disease [37].

### 4.4. Breast Milk Phospholipids and Proteins

Breast milk phospholipid concentrations were higher in the pet-exposed group. Higher phospholipid content may support early brain development, infant cognitive development, and the digestion, absorption, transport, and utilization of fatty acids [20]. The concentrations of PI 36:2 and PE 38:4 were significantly higher in the pet-exposed group than in the control group, and PI 36:2 (C18:0–C18:2, 0.0107%) was the most abundant PI molecule in breast milk. PE 38:4 contains arachidonic acid (AA, C20:4), an essential fatty acid in infancy that contributes to brain and nervous system development. Therefore, the higher breast milk phospholipid concentrations observed in the pet-exposed group may be relevant to infant neurodevelopment, although this study did not assess neurodevelopmental outcomes [38].

Maternal pet exposure was also associated with higher breast milk lysine concentrations. Lysine is involved in growth and nervous-system development and has been linked to infant growth-related outcomes [39]. Albanese et al. supplemented infants aged 1–27 weeks with lysine for 3–4 weeks [40]. These infants had greater weight gain, strength and stamina, and blood protein levels during the supplementation period [40]. Pereira et al. reported greater height gain in preschool children aged 2–5 years who received a lysine-containing flour diet daily for 6 months than in children without lysine supplementation [41]. These findings suggest that lysine may support growth in infants and children.

Breast milk β-casein concentrations were higher in mothers with pet exposure. β-Casein contains multiple serine and threonine residues that can be phosphorylated. β-Casein micelles can bind calcium ions and other divalent cations, thereby supporting calcium absorption and infant bone and nervous-system development [42]. β-Casein is readily digested by infants and is a major source of bioactive peptides in breast milk. Thus, β-casein-derived peptides may support neonatal gastrointestinal development [43].

### 4.5. Comparison with Previous Studies, Strengths, and Limitations

A previous study of pet-exposed adults aged 50–80 years reported lower inflammatory cytokine levels associated with depression, dementia, diabetes, cancer, and cardiovascular disease, but no differences in gut microbiota composition. This finding differs from the present results. This difference may reflect the marked changes in gut microbiota from early to late pregnancy, which are partly driven by hormonal shifts and are susceptible to internal and external factors [44]. The duration of pet–human contact may also affect microbiota transmission. To minimize the influence of transient microbial transmission, we included women with pet exposure before conception, during pregnancy, and postpartum, thereby capturing sustained pet–human contact. Continued follow-up of this cohort will allow for evaluation of associations between maternal pet exposure, gut microbiota, and childhood health outcomes, including allergies, asthma, and obesity, at approximately 3 years of age.

This study has several strengths. First, it used a prospective cohort with a clearly defined pet-exposure window spanning preconception, pregnancy, and the postpartum period. Second, it integrated maternal gut microbiota, breast milk microbial and nutritional profiles, and infant gut microbiota, with adjustment for potential confounders, including fermented milk intake.

Several limitations should also be noted. The single-center design and modest sample size limited the statistical power of subgroup analyses. The limited duration of infant follow-up prevented evaluation of long-term childhood outcomes, including allergic diseases and obesity. In addition, 16S rRNA sequencing provided limited taxonomic resolution, and targeted breast milk component analysis did not allow for species- or strain-level microbial identification or untargeted metabolomic profiling. Future multicenter studies should use refined pet-exposure assessments, larger cohorts, extended follow-up, mechanistic analyses, and integrated multiomics approaches to validate and extend these findings.

## 5. Conclusions

Our findings suggest that pet exposure before conception, during pregnancy, and postpartum was not associated with maternal GDM or GWG. However, pet exposure was associated with differences in selected bacterial taxa in the maternal and infant gut and with differences in breast milk composition. In the maternal gut, the pet-exposed group had higher relative abundances of Verrucomicrobia, Sutterellaceae, and *Akkermansia muciniphila* and a lower relative abundance of *Ruminococcus*, taxa that have been linked to metabolic health in previous studies. Pet exposure was also associated with higher breast milk concentrations of PI 36:2, PE 38:4, lysine, and β-casein, components that may be relevant to infant neurodevelopment and growth. The pet-exposed group also had a lower relative abundance of *Escherichia-Shigella* in the infant gut. Overall, these findings suggest that maternal pet exposure may be associated with maternal–infant microbial and breast milk compositional profiles that warrant further investigation in larger mechanistic studies.

## Figures and Tables

**Table 1:** Baseline characteristics of the study population according to pet exposure status.

	Overall	Pet Exposure Status (≥1 Household Pet)		
		Exposed	Unexposed	*p*-Value
	*n* (%)	*n* (%)	*n* (%)	
	54 (100)	22 (40.7%)	32 (59.3%)	
Pet	22		-	-
Dogs		14 (63.6)		
Cats		10 (45.5)		
Cats and dogs		2 (9.1)		
Maternal age at pregnancy				
<35 years	39 (72.2)	19 (48.7)	20 (51.3)	0.05
≥35 years	15 (27.8)	3 (20.0)	12 (80.0)	
Pre-pregnancy BMI				
Overweight	17	7	10	0.83
Obesity	34	13	21	
Fermented milk consumption				
No	15 (27.8)	5 (33.3)	10 (66.7)	0.04
Yes	39 (72.2)	17 (43.6)	22 (56.4)	
Birth mode				0.75
Vaginal	21 (38.9)	8 (38.1)	13 (61.9)	
Cesarean delivery	33 (61.1)	14 (42.4)	19 (57.6)	
Vaginitis during pregnancy				1.00
No	43 (93.5)	20 (46.5)	23 (53.5)	
Yes	3 (6.5)	1 (33.3)	2 (66.7)	
Urinary tract infection during pregnancy				0.45
No	45 (97.8)	20 (44.4)	25 (55.6)	
Yes	1 (2.2)	1 (100.0)	0 (0.0)	
Gestational diabetes mellitus				0.13
No	41 (75.9)	19 (46.3)	22 (53.7)	
Yes	13 (24.1)	3 (23.1)	10 (76.9)	
Maternal asthma during pregnancy				0.45
No	45 (97.8)	20 (44.4)	25 (55.6)	
Yes	1 (2.2)	1 (100.0)	0 (0.0)	
Maternal allergy during pregnancy				0.49
No	44 (95.7)	21 (47.7)	23 (52.3)	
Yes	2 (4.3)	0 (0.0)	2 (100.0)	
Maternal antibiotic exposure during pregnancy				1.00
No	45 (97.8)	21 (46.7)	24 (53.3)	
Yes	1 (2.2)	0 (0.0)	1 (100.0)	
Breastfeeding status				0.00
None	5 (10.9)	4 (80.0)	1 (20.0)	
Partial	17 (37.0)	8 (47.1)	9 (52.9)	
Exclusive	24 (52.1)	9 (37.5)	15 (62.5)	
Preterm birth				1.00
No	53 (98.1)	22 (41.5)	31 (58.5)	
Yes	1 (1.9)	0 (0.0)	1 (100.0)	

**Note:** “-” indicates no data available for this group.

**Table 2:** Lipid and glucose metabolic indices according to pet exposure and fermented milk consumption.

Metabolic Indicator (mmol/L)		Fermented Milk Consumption				No Fermented Milk Consumption			
		P-F (*n* = 16)	NP-F (*n* = 22)	*t*-Value	*p*-Value	P-NF (*n* = 5)	NP-NF (*n* = 8)	*t*-Value	*p*-Value
Blood lipids	TG	1.19 ± 0.30	1.28 ± 0.45	−0.71	0.48	1.30 ± 0.16	1.23 ± 0.26	0.63	0.54
	CHOL	4.70 ± 0.87	4.62 ± 1.13	0.22	0.82	4.38 ± 0.53	4.43 ± 0.58	−0.16	0.87
	HDL-C	1.52 ± 0.34	1.45 ± 0.28	0.67	0.51	1.30 ± 0.17	1.43 ± 0.13	−1.31	0.23
	LDL-C	2.80 ± 0.67	2.76 ± 0.92	0.15	0.89	2.71 ± 0.36	2.55 ± 0.56	0.58	0.58
Blood glucose	Fasting glucose	4.53 ± 0.46	4.77 ± 0.34	−1.83	0.08	4.94 ± 0.33	4.99 ± 0.37	−0.23	0.83
	OGTT fasting (0 h)	4.51 ± 0.50	4.59 ± 0.43	−0.49	0.63	4.34 ± 0.15	4.43 ± 0.36	−0.55	0.59
	OGTT 1 h	8.05 ± 1.24	8.27 ± 1.66	−0.46	0.65	7.52 ± 1.14	7.91 ± 2.13	−0.42	0.68
	OGTT 2 h	6.23 ± 1.12	6.88 ± 1.07	−1.73	0.09	6.57 ± 0.98	6.84 ± 1.51	−0.39	0.71

**Note:** Data are presented as mean ± SD. Abbreviations: P-F, pet exposure with fermented milk consumption; NP-F, no pet exposure with fermented milk consumption; P-NF, pet exposure without fermented milk consumption; NP-NF, no pet exposure without fermented milk consumption; TG, triacylglycerol; CHOL, cholesterol; HDL-C, high-density lipoprotein cholesterol; LDL-C, low-density lipoprotein cholesterol; OGTT, oral glucose tolerance test.

## Data Availability

The raw sequencing data generated in this study have been deposited in the Genome Sequence Archive [45] at the National Genomics Data Center [46], China National Center for Bioinformation/Beijing Institute of Genomics, Chinese Academy of Sciences. The dataset is publicly accessible at https://ngdc.cncb.ac.cn/gsa-human (accessed on 25 September 2025) under GSA-Human accession number HRA013429.

## Supplementary Materials

The following supporting information can be downloaded at https://www.mdpi.com/article/10.3390/metabo16050317/s1, Figure S1: Gestational weight gain in the P-F, NP-F, P-NF, and NP-NF groups. Figure S2: Alpha diversity indices (ACE, Chao1, and Shannon) of maternal gut microbiota in the P-F and NP-F groups. Figure S3: Alpha diversity indices (ACE, Chao1, and Shannon) of maternal gut microbiota in the P-NF and NP-NF groups. Figure S4: Alpha diversity indices (ACE, Chao1, and Shannon) of breast milk microbiota in the P and NP groups. Figure S5: Principal coordinate analysis based on unweighted UniFrac distances of breast milk microbiota. Figure S6: Alpha diversity indices (ACE, Chao1, and Shannon) of infant gut microbiota in the P and NP groups. Table S1: Relative abundance of dominant bacterial taxa in maternal stool during pregnancy and the postpartum period, stratified by pet exposure and fermented milk consumption. Table S2: Relative abundance of dominant bacterial taxa in maternal stool at all pregnancy stages, stratified by pet exposure and fermented milk consumption. Table S3: Relative abundance of dominant bacterial taxa in breast milk, stratified by pet exposure and fermented milk consumption.

## Abbreviations

The following abbreviations are used in this manuscript:

GWG	gestational weight gain
GDM	gestational diabetes mellitus
P	pet exposure
NP	no pet exposure
P-F	pet exposure with fermented milk consumption
NP-F	no pet exposure with fermented milk consumption
P-NF	pet exposure without fermented milk consumption
NP-NF	no pet exposure without fermented milk consumption
OTU	operational taxonomic unit
PC	phosphatidylcholine
PE	phosphatidylethanolamine
SM	sphingomyelin
PI	phosphatidylinositol
PG	phosphatidylglycerol
Cer	ceramides
TAG	triglyceride
UPLC	ultra-performance liquid chromatography
ACE	abundance-based coverage estimator
PCoA	principal coordinate analysis
KEGG	Kyoto Encyclopedia of Genes and Genomes
LDA	linear discriminant analysis
LEfSe	linear discriminant analysis effect dize
OGTT	oral glucose tolerance test
E/B ratio	the ratio of Enterobacteriaceae to Bacteroidaceae
LPS	lipopolysaccharide
LBP	lipopolysaccharide-binding protein
AA	arachidonic acid
