# Supplementary material for: Pet Exposure Is Associated with Altered Gut Microbiota and Higher Phospholipid and Protein Concentrations in the Breast Milk of Overweight/Obese Pregnant Women
Source: Metabolites. 2026 May 9;16(5):317. doi: 10.3390/metabo16050317 (PMC13208356; doi:10.3390/metabo16050317)
Supplement: Supplementary file 1 [file metabolites-16-00317-s001.zip › S Figure/Table_S1.docx]

| **Table S1.** Relative abundance of dominant bacterial taxa in maternal stool during pregnancy and the postpartum period, stratified by pet exposure pet and fermented milk consumption. | | | | | | | | | |
| --- | --- | --- | --- | --- | --- | --- | --- | --- | --- |
| Taxa | Overall | | | Fermented milk | | | No Fermented milk | | |
|  | Pet  22（40.7%）  Median（IQR） | No Pet  32（59.3%）  Median（IQR） | P | Pet  17（77.3%）  Median（IQR） | No Pet  5（22.7%）  Median（IQR） | P | Pet  22（68.8%）  Median（IQR） | No Pet  10（31.3%）  Median（IQR） | P |
| Actinobacteria | 0.03（0.07） | 0.03（0.06） | 0.21 | 0.03（0.07） | 0.04（0.10） | 0.92 | 0.03（0.06） | 0.02（0.02） | 0.10 |
| Bifidobacteriaceae | 0.03（0.07） | 0.02（0.06） | 0.54 | 0.03（0.07） | 0.03（0.08） | 0.72 | 0.02（0.03） | 0.01（0.02） | 0.21 |
| Bifidobacterium | 0.03（0.07） | 0.02（0.06） | 0.55 | 0.03（0.07） | 0.03（0.08） | 0.71 | 0.02（0.03） | 0.01（0.02） | 0.21 |
| Bacteroidetes | 0.37（0.27） | 0.36（0.35） | 0.73 | 0.34（0.24） | 0.34（0.35） | 0.81 | 0.40（0.38） | 0.40（0.35） | 0.45 |
| Bacteroidaceae | 0.17（0.18） | 0.18（0.24） | 0.49 | 0.17（0.17） | 0.17（0.19） | 0.80 | 0.19（0.23） | 0.25（0.34） | 0.26 |
| Bacteroides | 0.17（0.18） | 0.18（0.24） | 0.49 | 0.17（0.17） | 0.17（0.19） | 0.79 | 0.19（0.23） | 0.24（0.35） | 0.26 |
| Parabacteroides | 0.02（0.03） | 0.01（0.02） | 0.16 | 0.01（0.02） | 0.01（0.02） | 0.57 | 0.04（0.03） | 0.01（0.02） | 0.02* |
| Barnesiellaceae | 0.003（0.01） | 0.003（0.01） | 0.11 | 0.003（0.01） | 0.002（0.007） | 0.01* | 0.005（0.02） | 0.004（0.02） | 0.74 |
| Barnesiella | 0.003（0.01） | 0.003（0.01） | 0.11 | 0.003（0.01） | 0.002（0.007） | 0.01* | 0.004（0.02） | 0.004（0.01） | 0.73 |
| Prevotellaceae | 0.03（0.10） | 0.01（0.07） | 0.15 | 0.05（0.12） | 0.02（0.17） | 0.06 | 0.01（0.02） | 0.01（0.02） | 0.25 |
| Prevotella | 0.02（0.06） | 0.01（0.04） | 0.27 | 0.04（0.08） | 0.01（0.15） | 0.17 | 0.005（0.01） | 0.008（0.02） | 0.61 |
| Rikenellaceae | 0.01（0.02） | 0.01（0.02） | 0.33 | 0.01（0.02） | 0.01（0.02） | 0.09 | 0.02（0.04） | 0.02（0.03） | 0.93 |
| Alistipes | 0.01（0.02） | 0.01（0.02） | 0.35 | 0.01（0.02） | 0.01（0.02） | 0.09 | 0.02（0.04） | 0.02（0.03） | 0.86 |
| Firmicutes | 0.44（0.20） | 0.43（0.22） | 0.87 | 0.44（0.19） | 0.48（0.19） | 0.75 | 0.45（0.21） | 0.41（0.25） | 0.99 |
| Streptococcaceae | 0.01（0.01） | 0.00（0.01） | 0.92 | 0.01（0.01） | 0.01（0.02） | 0.62 | 0.00（0.01） | 0.00（0.00） | 0.73 |
| Streptococcus | 0.01（0.01） | 0.00（0.01） | 0.91 | 0.01（0.01） | 0.01（0.02） | 0.61 | 0.003（0.01） | 0.003（0.005） | 0.69 |
| Clostridiaceae | 0.002（0.003） | 0.002（0.003） | 0.52 | 0.00（0.001） | 0.00（0.003） | 0.42 | 0.004（0.005） | 0.002（0.002） | 0.03* |
| Clostridium_sensu_stricto_1 | 0.001（0.003） | 0.002（0.003） | 0.67 | 0.002（0.002） | 0.003（0.003） | 0.36 | 0.004（0.005） | 0.002（0.002） | 0.045* |
| Subdoligranulum | 0.02（0.02） | 0.01（0.01） | 0.009** | 0.02（0.02） | 0.01（0.02） | 0.08 | 0.02（0.01） | 0.01（0.01） | 0.09 |
| Faecalibacterium | 0.05（0.08） | 0.04（0.05） | 0.37 | 0.06（0.10） | 0.05（0.07） | 0.31 | 0.04（0.03） | 0.03（0.03） | 0.93 |
| Megamonas | 0.001（0.003） | 0.001（0.005） | 0.07 | 0.001（0.003） | 0.001（0.005） | 0.76 | 0.001（0.001） | 0.002（0.004） | 0.003* |
| Lachnospiraceae | 0.16（0.11） | 0.16（0.12） | 0.82 | 0.17（0.11） | 0.18（0.14） | 0.69 | 0.16（0.14） | 0.15（0.11） | 0.95 |
| Fusicatenibacter | 0.007（0.007） | 0.006（0.01） | 0.18 | 0.007（0.01） | 0.007（0.01） | 0.29 | 0.006（0.005） | 0.005（0.007） | 0.61 |
| Agathobacter | 0.01（0.02） | 0.01（0.02） | 0.45 | 0.01（0.02） | 0.02（0.03） | 0.48 | 0.01（0.01） | 0.01（0.01） | 0.76 |
| Blautia | 0.03（0.03） | 0.03（0.05） | 0.81 | 0.03（0.03） | 0.04（0.09） | 0.36 | 0.04（0.04） | 0.02（0.02） | 0.08 |
| Coprococcus | 0.003（0.004） | 0.003（0.005） | 0.63 | 0.003（0.003） | 0.003（0.004） | 0.62 | 0.004（0.004） | 0.004（0.006） | 0.12 |
| Ruminococcaceae | 0.10（0.11） | 0.09（0.09） | 0.26 | 0.16（0.12） | 0.09（0.09） | 0.048* | 0.08（0.05） | 0.09（0.09） | 0.24 |
| Ruminococcus | 0.005（0.009） | 0.006（0.009） | 0.30 | 0.005（0.01） | 0.005（0.005） | 0.62 | 0.004（0.005） | 0.01（0.01） | 0.005** |
| Veillonellaceae | 0.01（0.02） | 0.01（0.02） | 0.23 | 0.02（0.03） | 0.01（0.02） | 0.06 | 0.00（0.01） | 0.01（0.01） | 0.56 |
| Veillonella | 0.00（0.001） | 0.00（0.00） | 0.74 | 0.00（0.002） | 0.00（0.001） | 0.34 | 0.00（0.00） | 0.00（0.001） | 0.028* |
| Dialister | 0.01（0.02） | 0.01（0.01） | 0.17 | 0.02（0.02） | 0.01（0.01） | 0.06 | 0.02（0.01） | 0.03（0.01） | 0.84 |
| Peptostreptococcaceae | 0.009（0.009） | 0.007（0.008） | 0.07 | 0.01（0.01） | 0.01（0.01） | 0.33 | 0.01（0.01） | 0.01（0.01） | 0.08 |
| Selenomonadaceae | 0.001（0.003） | 0.001（0.005） | 0.11 | 0.001（0.003） | 0.001（0.004） | 0.67 | 0.001（0.002） | 0.002（0.004） | 0.03* |
| Sutterellaceae | 0.008（0.02） | 0.003（0.01） | <0.001** | 0.01（0.02） | 0.003（0.01） | 0.001** | 0.009（0.02） | 0.002（0.005） | 0.01* |
| Proteobacteria | 0.03（0.04） | 0.03（0.03） | 0.007** | 0.03（0.04） | 0.03（0.02） | 0.09 | 0.04（0.04） | 0.02（0.03） | 0.02* |
| Enterobacteriaceae | 0.01（0.02） | 0.01（0.002） | 0.96 | 0.01（0.01） | 0.01（0.02） | 0.17 | 0.02（0.02） | 0.01（0.01） | 0.04* |
| Escherichia-Shigella | 0.004（0.01） | 0.005（0.02） | 0.62 | 0.003（0.005） | 0.007（0.02） | 0.12 | 0.01（0.02） | 0.00（0.01） | 0.17 |
| Pasteurellaceae | 0.00（0.001） | 0.00（0.002） | 0.67 | 0.00（0.001） | 0.00（0.001） | 0.69 | 0.001（0.001） | 0.001（0.002） | 0.24 |
| Haemophilus | 0.00（0.001） | 0.00（0.001） | 0.74 | 0.00（0.002） | 0.00（0.002） | 0.75 | 0.00（0.001） | 0.00（0.002） | 0.35 |
| Verrucomicrobiota | 0.00（0.002） | 0.00（0.001） | 0.55 | 0.00（0.00） | 0.00（0.00） | 0.29 | 0.00（0.00） | 0.00（0.00） | 0.03* |
| Akkermansiaceae | 0.00（0.002） | 0.00（0.001） | 0.49 | 0.00（0.002） | 0.001（0.002） | 0.31 | 0.001（0.003） | 0.00（0.00） | 0.03* |
| Akkermansia muciniphila | 0.00（0.002） | 0.00（0.001） | 0.49 | 0.00（0.002） | 0.001（0.002） | 0.31 | 0.001（0.003） | 0.00（0.00） | 0.03* |
| Values showed the median abundance of maternal gut microbiota filtered by p-value (“*” stands for p-value less than 0.05; “**” stands for p-value less than 0.01) and false discovery rate (FDR, less than 0.1) for multiple comparisons. | | | | | | | | | |
