# Supplementary material for: Pet Exposure Is Associated with Altered Gut Microbiota and Higher Phospholipid and Protein Concentrations in the Breast Milk of Overweight/Obese Pregnant Women
Source: Metabolites. 2026 May 9;16(5):317. doi: 10.3390/metabo16050317 (PMC13208356; doi:10.3390/metabo16050317)
Supplement: Supplementary file 1 [file metabolites-16-00317-s001.zip › S Figure/Table_S2.docx]

| **Table S2.** Relative abundance of dominant bacterial taxa in maternal stool at all stages of pregnancy, stratified by pet exposure and fermented milk consumption. | | | | | | | | | |
| --- | --- | --- | --- | --- | --- | --- | --- | --- | --- |
| Taxa | early pregnancy | | | pregnant metaphase | | | Late pregnancy | | |
|  | Pet  22（40.7%）  Media（IQR） | No Pet  32（59.3%）  Media（IQR） | P | Pet  22（40.7%）  Media（IQR） | No Pet  32（59.3%）  Media（IQR） | P | Pet  22（40.7%）  Media（IQR） | No Pet  32（59.3%）  Media（IQR） | P |
| Actinobacteria | 0.03（0.03） | 0.03（0.07） | 0.27 | 0.03（0.07） | 0.05（0.10） | 0.80 | 0.03（0.06） | 0.03（0.05） | 0.99 |
| Bifidobacteriaceae | 0.02（0.02） | 0.03（0.07） | 0.16 | 0.03（0.07） | 0.03（0.11） | 0.75 | 0.02（0.05） | 0.02（0.05） | 0.99 |
| Bifidobacterium | 0.02（0.02） | 0.03（0.07） | 0.16 | 0.03（0.07） | 0.03（0.11） | 0.75 | 0.02（0.05） | 0.02（0.05） | 0.99 |
| Bacteroidetes | 0.34（0.26） | 0.47（0.29） | 0.51 | 0.47（0.29） | 0.34（0.42） | 0.28 | 0.38（0.11） | 0.39（0.35） | 0.86 |
| Bacteroidaceae | 0.16（0.17） | 0.23（0.21） | 0.77 | 0.23（0.21） | 0.14（0.22） | 0.26 | 0.21（0.13） | 0.21（0.16） | 0.75 |
| Bacteroides | 0.16（0.17） | 0.23（0.21） | 0.77 | 0.23（0.21） | 0.14（0.22） | 0.26 | 0.21（0.13） | 0.21（0.16） | 0.75 |
| Firmicutes | 0.52（0.27） | 0.41（0.16） | 0.19 | 0.41（0.16） | 0.43（0.19） | 0.38 | 0.41（0.15） | 0.42（0.27） | 0.75 |
| Streptococcaceae | 0.01（0.01） | 0.00（0.01） | 0.96 | 0.003（0.008） | 0.004（0.01） | 0.61 | 0.005（0.006） | 0.005（0.012） | 0.56 |
| Streptococcus | 0.01（0.01） | 0.00（0.01） | 0.96 | 0.003（0.008） | 0.004（0.01） | 0.57 | 0.01（0.01） | 0.00（0.01） | 0.66 |
| Clostridiaceae | 0.002（0.002） | 0.002（0.003） | 0.33 | 0.002（0.003） | 0.002（0.003） | 0.61 | 0.002（0.003） | 0.002（0.003） | 0.77 |
| Clostridium | 0.002（0.002） | 0.002（0.003） | 0.46 | 0.002（0.003） | 0.002（0.003） | 0.96 | 0.001（0.003） | 0.002（0.003） | 0.83 |
| Lachnospiraceae | 0.22（0.13） | 0.15（0.10） | 0.07 | 0.15（0.10） | 0.19（0.11） | 0.06 | 0.15（0.08） | 0.14（0.13） | 0.80 |
| Blautia | 0.05（0.04） | 0.03（0.03） | 0.19 | 0.03（0.03） | 0.04（0.07） | 0.29 | 0.03（0.02） | 0.02（0.04） | 0.73 |
| Coprococcus | 0.005（0.004） | 0.004（0.002） | 0.51 | 0.004（0.002） | 0.004（0.004） | 0.91 | 0.003（0.003） | 0.004（0.005） | 0.49 |
| Ruminococcaceae | 0.13（0.10） | 0.11（0.10） | 0.55 | 0.11（0.10） | 0.01（0.08） | 0.15 | 0.10（0.18） | 0.10（0.10） | 0.27 |
| Veillonellaceae | 0.01（0.02） | 0.02（0.02） | 0.80 | 0.02（0.02） | 0.01（0.01） | 0.18 | 0.02（0.03） | 0.01（0.02） | 0.13 |
| Veillonella | 0.00（0.001） | 0.00（0.001） | 0.42 | 0.00（0.001） | 0.00（0.001） | 0.64 | 0.00（0.001） | 0.00（0.001） | 0.91 |
| Proteobacteria | 0.03 | 0.03 | 0.51 | 0.03（0.04） | 0.03（0.03） | 0.40 | 0.04（0.04） | 0.03（0.03） | 0.31 |
| Enterobacteriaceae | 0.01（0.01） | 0.01（0.01） | 0.62 | 0.008（0.01） | 0.007（0.02） | 0.96 | 0.01（0.01） | 0.01（0.01） | 0.18 |
| Escherichia-Shigella | 0.01（0.01） | 0.00（0.01） | 0.93 | 0.005（0.01） | 0.003（0.01） | 0.69 | 0.003（0.004） | 0.005（0.005） | 0.33 |
| Pasteurellaceae | 0.001（0.001） | 0.001（0.002） | 0.97 | 0.001（0.002） | 0.001（0.002） | 0.80 | 0.001（0.002） | 0.001（0.004） | 0,98 |
| Haemophilus | 0.001（0.001） | 0.001（0.001） | 1 | 0.001（0.001） | 0.002（0.002） | 0.81 | 0.001（0.001） | 0.001（0.003） | 0.94 |
| Verrucomicrobia | 0.001（0.001） | 0.001（0.002） | 0.55 | 0.001（0.002） | 0.000（0.001） | 0.28 | 0.001（0.001） | 0.000（0.001） | 0.45 |
