# Supplementary material for: Pet Exposure Is Associated with Altered Gut Microbiota and Higher Phospholipid and Protein Concentrations in the Breast Milk of Overweight/Obese Pregnant Women
Source: Metabolites. 2026 May 9;16(5):317. doi: 10.3390/metabo16050317 (PMC13208356; doi:10.3390/metabo16050317)
Supplement: Supplementary file 1 [file metabolites-16-00317-s001.zip › S Figure/Table_S3.docx]

| **Table S3.** Relative abundance of dominant bacterial taxa in breast milk, stratified by pet exposure and fermented milk consumption. | | | | | | | | | | |
| --- | --- | --- | --- | --- | --- | --- | --- | --- | --- | --- |
| Taxa | Overall | | | | Probiotics | |  | No Probiotics | |  |
|  | Pet  22（40.7%）  Media（IQR） | | No Pet  32（59.3%）  Media（IQR） | P | Pet  17（77.3%）  Media（IQR） | No Pet  5（22.7%）  Media（IQR） | P | Pet  22（68.8%）  Media（IQR） | No Pet  10（31.3%）  Media（IQR） | P |
| Actinobacteria | 0.01（0.01） | 0.01（0.04） | | 0.26 | 0.006（0.01） | 0.009（0.02） | 0.57 | 0.01（0.01） | 0.01（0.06） | 0.28 |
| Acinetobacter | 0.02（0.36） | 0.02（0.05） | | 0.14 | 0.02（0.20） | 0.02（0.05） | 0.31 | 0.04（0.57） | 0.01（0.06） | 0.36 |
| Bifidobacterium | 0.00（0.001） | 0.00（0.002） | | 0.66 | 0.00（0.001） | 0.00（0.002） | 0.93 | 0.001（0.002） | 0.00（0.00） | 0.24 |
| Corynebacterium | 0.00（0.001） | 0.001（0.002） | | 0.14 | 0.00（0.001） | 0.001（0.001） | 0.43 | 0.00（0.001） | 0.001（0.003） | 0.11 |
| Rothia | 0.00（0.001） | 0.001（0.002） | | 0.04* | 0.00（0.001） | 0.001（0.002） | 0.25 | 0.00（0.00） | 0.001（0.01） | 0.05 |
| Bacteroidetes | 0.01（0.04） | 0.01（0.06） | | 0.84 | 0.003（0.02） | 0.008（0.07） | 0.38 | 0.02（0.09） | 0.02（0.05） | 0.45 |
| Bacteroides | 0.001（0.009） | 0.00（0.002） | | 0.58 | 0.00（0.00） | 0.00（0.02） | 0.79 | 0.003（0.04） | 0.00（0.002） | 0.22 |
| Staphylococcus | 0.04（0.10） | 0.05（0.27） | | 0.66 | 0.04（0.14） | 0.04（0.31） | 0.57 | 0.07（0.09） | 0.06（0.16） | 0.84 |
| Streptococcus | 0.02（0.05） | 0.04（0.25） | | 0.08 | 0.01（0.05） | 0.02（0.11） | 0.20 | 0.02（0.03） | 0.17（0.34） | 0.09 |
| Lactobacillus | 0.002（0.003） | 0.002（0.008） | | 0.52 | 0.002（0.002） | 0.001（0.008） | 0.49 | 0.002（0.006） | 0.002（0.006） | 0.90 |
| Prevotella | 0.00（0.002） | 0.001（0.004） | | 0.26 | 0.00（0.001） | 0.001（0.005） | 0.17 | 0.001（0.003） | 0.001（0.003） | 0.97 |
| Firmicutes | 0.13（0.61） | 0.44（0.59） | | 0.41 | 0.37（0.60） | 0.38（0.63） | 1.00 | 0.12（0.48） | 0.58（0.60） | 0.13 |
| Faecalibacterium | 0.00（0.002） | 0.00（0.003） | | 0.83 | 0.00（0.001） | 0.001（0.01） | 0.36 | 0.002（0.006） | 0.00（0.002） | 0.27 |
| Pediococcus | 0.00（0.00） | 0.00（0.00） | | 0.01* | 0.00（0.00） | 0.00（0.00） | 0.09 | 0.00（0.00） | 0.00（0.00） | 0.06 |
| Pseudomonas | 0.005（0.02） | 0.005（0.02） | | 0.84 | 0.005（0.009） | 0.006（0.09） | 0.35 | 0.00（0.00） | 0.00（0.00） | 1 |
| Serratia | 0.003（0.01） | 0.005（0.008） | | 0.70 | 0.07（0.01） | 0.08（0.03） | 0.85 | 0.001（0.003） | 0.002（0.004） | 0.65 |
| Proteobacteria | 0.63（0.69） | 0.24（0.77） | | 0.29 | 0.40（0.77） | 0.27（0.79） | 1.00 | 0.77（0.51） | 0.08（0.71） | 0.05 |
| Escherichia_Shigella | 0.002（0.006） | 0.00（0.004） | | 0.53 | 0.00（0.003） | 0.00（0.006） | 0.40 | 0.01（0.02） | 0.00（0.00） | 0.03* |
| Sphingomonas | 0.001（0.002） | 0.001（0.001） | | 0.50 | 0.001（00.002） | 0.00（0.001） | 0.61 | 0.001（0.002） | 0.001（0.001） | 0.72 |
| Pantoea | 0.001（0.003） | 0.001（0.002） | | 0.97 | 0.001（0.004） | 0.001（0.005） | 0.37 | 0.001（0.003） | 0.00（0.002） | 0.18 |
| Neisseria | 0.00（0.001） | 0.00（0.001） | | 0.90 | 0.00（0.003） | 0.00（0.002） | 0.84 | 0.00（0.00） | 0.00（0.001） | 0.90 |
| Ralstonia | 0.00（0.00） | 0.00（0.001） | | 0.88 | 0.00（0.00） | 0.00（0.001） | 0.88 | 0.00（0.00） | 0.00（0.00） | 0.93 |
| Verrucomicrobia | 0.00（0.00） | 0.00（0.00） | | 0.67 | 0.00（0.00） | 0.00（0.001） | 0.57 | 0.00（0.00） | 0.00（0.00） | 1 |
| Akkermansia | 0.00（0.00） | 0.00（0.00） | | 0.36 | 0.00（0.00） | 0.00（0.00） | 0.22 | 0.00（0.00） | 0.00（0.00） | 0.97 |
