# Supplementary figures and images for: Pet Exposure Is Associated with Altered Gut Microbiota and Higher Phospholipid and Protein Concentrations in the Breast Milk of Overweight/Obese Pregnant Women
Source: Metabolites. 2026 May 9;16(5):317. doi: 10.3390/metabo16050317 (PMC13208356; doi:10.3390/metabo16050317)

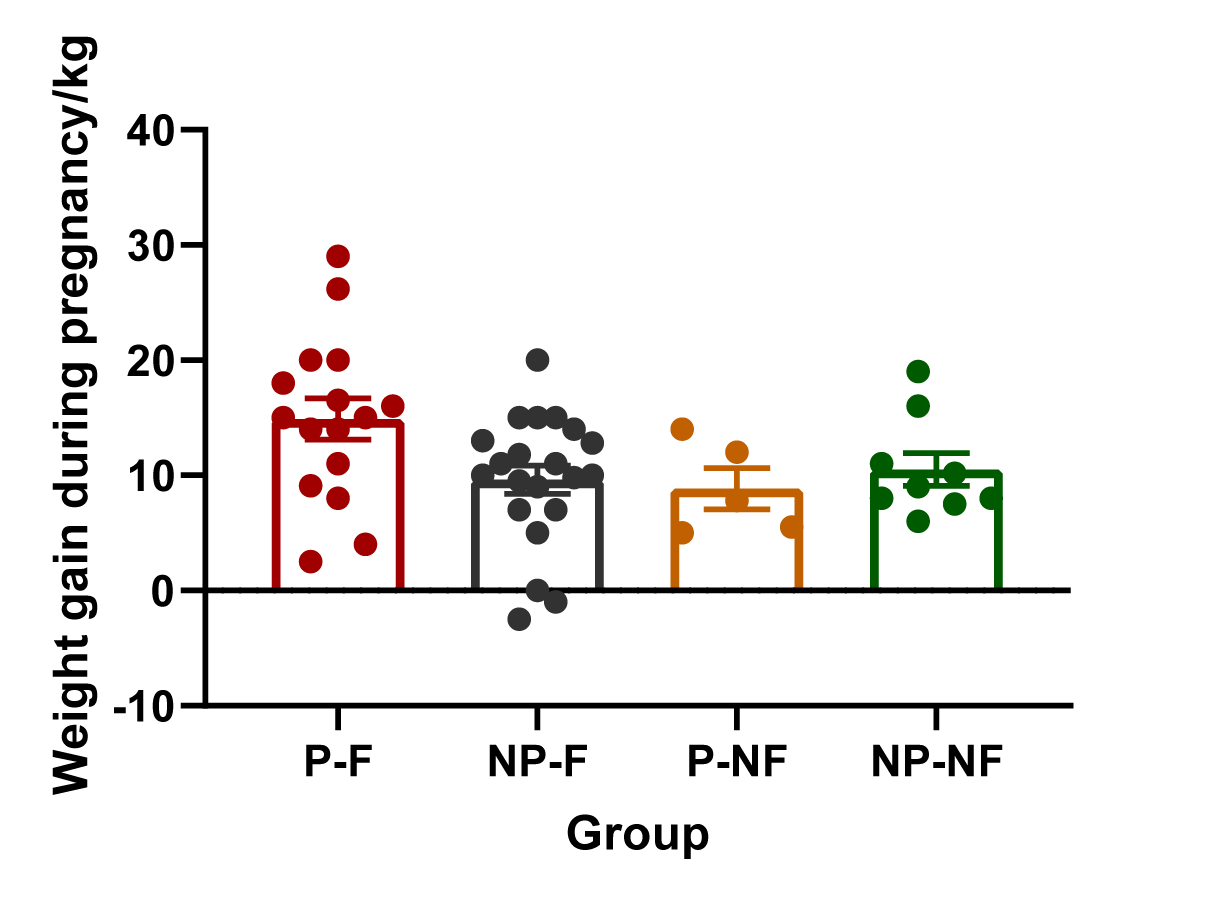

Supplement: Supplementary file 1 [file metabolites-16-00317-s001.zip › S Figure/Fig_S1.tif]

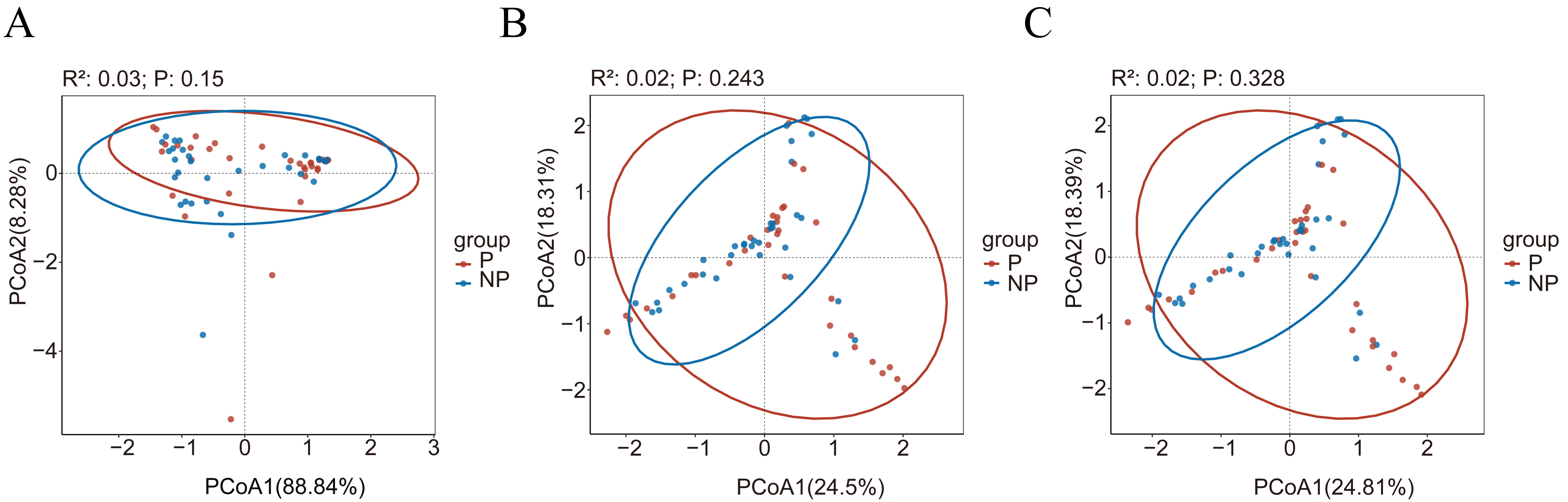

Supplement: Supplementary file 1 [file metabolites-16-00317-s001.zip › S Figure/Fig_S5.tif]
